# Food Intolerance and Allergy: Do They Have an Etiological Role in Idiopathic Granulomatous Mastitis?

**DOI:** 10.3390/jcm14030940

**Published:** 2025-02-01

**Authors:** Muge Yurdacan, Berrin Papila, Basar Can Turgut, Hafize Uzun, Mehmet Velidedeoglu

**Affiliations:** 1 Department of General Surgery, Bakirkoy Dr. Sadi Konuk Training and Research Hospital, Health Sciences University, 34147 Istanbul, Turkey; 2 Department of General Surgery, Cerrahpasa Medical Faculty, Istanbul University-Cerrahpasa, 34153 Istanbul, Turkey; papilaberrin@yahoo.com (B.P.); m-veli@hotmail.com (M.V.); 3 Department of General Surgery, Istanbul Training and Research Hospital, Health Sciences University, 34098 Istanbul, Turkey; basarcanturgut@gmail.com; 4 Department of Medical Biochemistry, Faculty of Medicine, Istanbul Atlas University, 34203 Istanbul, Turkey; huzun59@hotmail.com

**Keywords:** idiopathic granulomatous mastitis, food intolerance, histamine, interleukin-4, autoimmunity

## Abstract

**Background/Objectives:** Despite its long-standing recognition, the etiopathogenesis of idiopathic granulomatous mastitis (IGM) remains poorly understood. This study aims to investigate the relationship between IGM and food intolerance, allergies, and immunological factors to shed light on its etiology. **Materials and Methods:** This case–control study included 32 patients with IGM and 32 healthy women. In order to examine their potential relevance to allergy and immunology, serum interleukin (IL)-4, IL-4 receptor, histamine, and histamine-releasing factor (HRF) were measured by ELISA. Furthermore, serum IgG antibodies against specific food allergens were measured to evaluate food intolerance. **Results:** The patient group exhibited significantly higher intolerance values for lentils and curry compared to the control group (*p* = 0.023 and *p* = 0.012, respectively). Histamine (*p* < 0.001) and IL-4 (*p* = 0.003) levels were elevated in IGM patients compared to the control group, while HRF and IL-4R outcomes did not show any significant differences (*p* > 0.05). **Conclusions:** Elevated histamine and IL-4 levels may suggest the involvement of allergy and immunological factors in IGM’s etiopathogenesis. The integration of anti-histamine medications for IGM patients with elevated histamine levels could provide an alternative therapeutic strategy.

## 1. Introduction

Idiopathic granulomatous mastitis (IGM), initially delineated by Kessler and Woolloch in 1972, is an uncommon, chronic inflammatory breast disease that can cause severe painful symptoms and can mimic carcinoma clinically and radiologically [1]. Although precise prevalence and incidence figures are lacking, IGM appears to be more prevalent among Middle Eastern and Hispanic populations [2]. IGM can manifest at various ages, from 11 to 83 years old, but predominantly affects individuals during their reproductive years and shortly after pregnancy [3]. Notably, the majority of patients have a history of childbirth and lactation [4].

Despite considerable research, the precise etiology of IGM remains uncertain. Several hypotheses have been proposed, including those concerning autoimmune responses, infections, and hormonal factors [5]. Additionally, factors such as smoking, alpha1-antitrypsin deficiency, and oral contraceptive use have been suggested but not conclusively proven to be associated with IGM’s etiology. The positive responses to steroid and immunosuppressive treatments [6], the presence of extramammary findings such as erythema nodosum (EN) and arthritis, and the elevated T lymphocytes observed in immunohistochemical studies have collectively contributed to the growing notion that IGM may be an autoimmune disorder [5,7].

The clinical presentation of IGM typically involves a sudden onset of a painful, palpable breast mass accompanied by skin cutaneous redness. In chronic cases, additional manifestations may be observed, including bilateral pretibial painful panniculitis (erythema nodosum), fistulae, and axillary lymphadenopathy [8,9]. The definitive diagnosis necessitates pathological examination, as the clinical and radiological findings are insufficient in isolation [10]. The enigmatic nature of IGM’s etiology has hindered the establishment of standardized treatment protocols. Consequently, recent research has prioritized the investigation of etiology, particularly with regard to autoimmune factors. Remarkably, the prevalence of autoimmune diseases has significantly risen over the past three decades, which is attributed to environmental factors, especially diet. Recent studies have investigated the correlation between food intolerance, allergy, and autoimmune diseases, yielding valuable insights [11].

The present study was designed to investigate towards diet as prominent environmental factors contributing to the etiology of IGM. To this end, the objective was to conduct a study that would scrutinize the link between food intolerance and IGM by quantifying specific immunoglobulin-G (IgG) responses against 54 food antigens. Furthermore, our aim was to elucidate the correlation between IGM, allergy, and immunological factors through the assessment of interleukin-4 (IL-4), IL-4 receptor (IL-4R), histamine, and histamine-releasing factor (HRF). A comprehensive understanding of the etiology and pathogenesis of IGM could help identify high-risk groups and guide the development of appropriate treatment strategies.

## 2. Materials and Methods

### 2.1. Study Participants

This study, approved by the Clinical Trials Ethics Committee of the Istanbul University-Cerrahpaşa, Cerrahpasa Faculty of Medicine (No: 117806; date: 10 September 2020), adhered to the principles outlined in the Declaration of Helsinki. Written informed consent was obtained from both the patient and control groups following comprehensive verbal explanations.

- The study included 32 female patients with biopsy-confirmed IGM, who had not used corticosteroid or immunosuppressive medications in the previous year.
- The control group consisted of 32 healthy women, matched in age to the patient group, and with a history of childbirth and breastfeeding.

### 2.2. Procedure for Collection and Processing of Blood Samples

Following a 12 h fast, venous blood samples were collected from both groups in anticoagulant-free tubes. Subsequently, centrifugation at 3000× *g* for 15 min enabled the separation of plasma and serum. The serum samples were transferred to Eppendorf tubes and stored at −80 °C for later analysis.

### 2.3. Measurement of Serum IgG Antibodies Against Specific Food Allergens

A food intolerance kit comprising test strips coated with 54 different foods and food additives was used (egg white, egg yolk, cow’s milk, beta-lactoglobulin, casein, processed cheese, goat’s milk, wheat flour, rye flour, barley flour, oat flour, rice, buckwheat flour, soybean, corn, yeast, cod, tuna, salmon, prawn, beef, pork, chicken, orange, apple, kiwi, banana, fig, grape, peanut, hazelnut, almond, walnut, tomato, potato, carrot, white bean, green bean, lentil, garlic, onion, leek, cabbage, broccoli, mushroom, vanilla, curry, black pepper, cocoa, coffee, mustard, black tea, gluten, and honey). The serum samples were incubated with the test strips. If specific IgG and IgE class antibodies were present in the sample, they bound to the antigenic components on the strip. The bound antibodies were then detected via a secondary incubation using enzyme-labeled anti-human IgG. The EUROLINE-FOOD Profil 54 (IgG) kit (cat. no: DP30220802-1G, EUROIMMUN Medizinische Labordiagnostika AG, Lübeck, Germany) was used to measure food intolerance values as per the manufacturer’s instructions.

### 2.4. Measurement of Serum IL-4, IL-4R, Histamine, and HRF

The serum concentrations of IL-4 (cat. no: SEA077HU, Cloud-Clone Corp., Katy, TX, USA), IL-4 receptor (cat. no: SEC031Hu), histamine (cat. no: CEA927Ge, Cloud-Clone Corp., Katy, TX, USA), and HRF (cat. no: SEG962Hu, Cloud-Clone Corp., Katy, TX, USA) were measured using human enzyme-linked immunosorbent assay (ELISA) kits from Cloud-Clone Corp., Katy, TX, USA. The procedures were conducted as per the manufacturer’s instructions.

### 2.5. Statistical Analysis

Statistical analysis was conducted using SPSS 17.0. The normality of the variables was evaluated through the utilization of histogram graphics and the Kolmogorov–Smirnov test. Categorical variables were compared using the Pearson Chi-Square Test, while non-normally distributed (nonparametric) variables between the two groups were evaluated using the Mann–Whitney U Test. A *p*-value of less than 0.05 was considered statistically significant.

Using G*Power 3.1.2 software, a power analysis based on independent two-group statistics was performed. The analysis was conducted with Cohen’s medium effect size of 0.5, a Type I error rate (α) of 0.05, and a target power (1-β) of 0.80. Based on these parameters, the minimum required sample size was calculated to be n = 24 for each group, with a total of n = 48 participants. Accordingly, in our study, we included 32 participants in both the patient and control groups.

## 3. Results

The mean age of the 32 patients at the time of diagnosis was 35.16 ± 5.61 years (range: 22–45 years), while the mean age of the control group was 34.91 ± 7.19 years. No statistically significant difference was observed between the two groups in age distribution (*p* > 0.05). The mean body mass index (BMI) was recorded as 28.5 kg/m^2^.

Seven patients presented with erythema nodosum, with lesions appearing approximately two weeks after the onset of mastitis. Concurrent EN and mastitis were identified in only one patient. Six patients in the cohort were smokers, and a further six had chronic conditions such as hypothyroidism, asthma, and hypertension. Furthermore, three patients had a history of oral contraceptive use.

All cases were parous (median: 2.75) and had a history of breastfeeding (mean: 45 months; range: 6–102). Two patients exhibited IGM during the breastfeeding period, while one case manifested during pregnancy. The interval between the cessation of breastfeeding and the onset of mastitis ranged from zero to seven years, with a median of three years.

The symptoms and clinical findings of the IGM patients are presented in Table 1. Upon presentation to the clinic, the most prevalent complaint was the presence of a breast mass (84.38%), followed by pain (78.13%). Bilateral involvement was noted in five cases (15.6%), with simultaneous mastitis affecting both breasts in two of these patients (Table 1).

The median interval between symptom onset and hospital admission was 4.5 months. Recurrence was observed in a total of seven patients.

**Figure 1:**
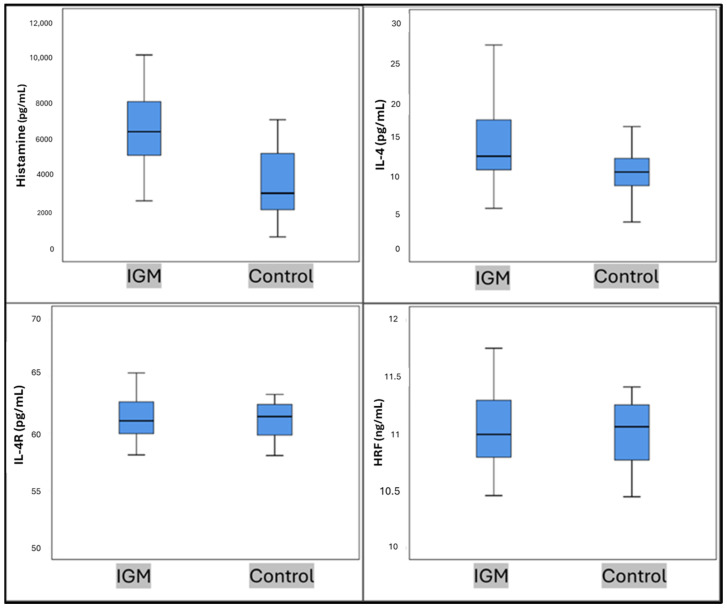
Histamine, IL-4, IL-4R, and HRF values in IGM and control groups. Mann–Whitney U Test.

A comparison was made between the values of histamine, IL-4, IL-4R, and HRF in both patient and control groups. Statistically significant elevations in histamine and IL-4 levels were observed within the IGM group in comparison to the control group (*p* < 0.01 and *p* = 0.03) (Table 2 and Figure 1).

**Figure 2:**
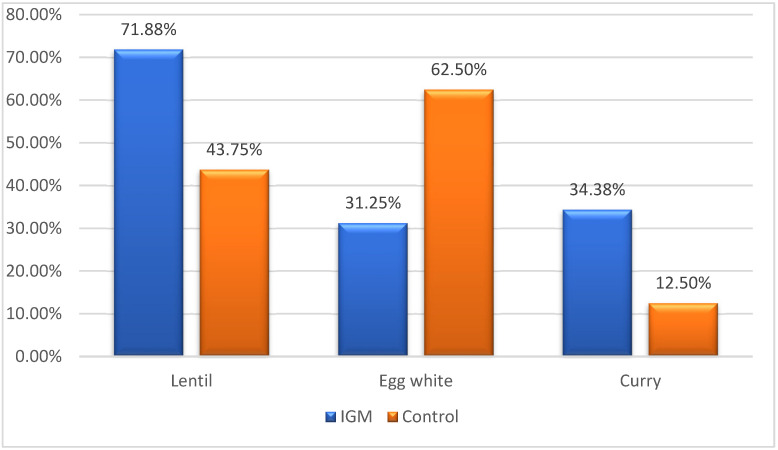
Significant food intolerance differences between patient and control groups.

Furthermore, IgG antibody values targeting 54 food antigens were quantified in both groups to evaluate sensitization to these food items and additives. In accordance with the instructions provided with the kit, an IgG level exceeding 12.5 was deemed indicative of sensitization. The prevalence of food intolerance was significantly higher in the patient cohort, with a notable increase in individuals reporting intolerance to lentils and curry (*p* = 0.023 and *p* = 0.039) (Figure 2 and Table 3). In addition, the data were reanalyzed using the Bonferroni correction to account for the effect of multiple comparisons. After this correction, the adjusted *p*-values for lentils and curry were 0.0051 and 0.0124, respectively, which remained below the adjusted significance threshold and maintained statistical significance.

The patient group received corticosteroid therapy at a dose of 0.5 mg/kg/day for 6 weeks. The treatment response was assessed by measuring the size of the mass. In patients with partial or complete response, the medication was tapered by reducing the dose by 5 mg per week at the end of the 6th week. Six of our patients exhibited steroid resistance. Methotrexate monotherapy (15 mg/week administered orally for 6 months) was prescribed to these patients. In the comparison of serum markers between corticosteroid-resistant and corticosteroid-sensitive patients, no statistically significant differences were observed. Histamine levels showed no significant difference (*p* = 0.531), while IL-4 levels (*p* = 0.866) and HRF and IL-4R levels (*p* = 0.128) did not differ significantly either.

## 4. Discussion

Idiopathic granulomatous mastitis is a rare, benign, chronic inflammatory disease. Its resemblance to breast cancer both radiologically and clinically necessitates careful consideration in the differential diagnosis [12]. The predominant presenting symptoms of IGM include a palpable unilateral breast mass, redness, nipple retraction, skin ulcers, discharge, and axillary masses [13]. Palpable masses are typically the most prevalent clinical findings [14,15]. In total, 84.4% of our patients presented with complaints of a palpable breast mass, which is consistent with the rates reported in the existing literature [15,16,17]. It is noteworthy that our study revealed a significant fistula rate of 43.8%. Although relatively high, this rate is similar to that reported by Alihassi et al.’s, who observed a fistula rate of 50% in their patients [14].

IGM’s development mechanism is thought to involve ductal epithelial damage, the migration of luminal secretions to lobular connective tissue, and the ensuing local granulomatous inflammatory response. Nevertheless, the factors that trigger this ductal epithelial damage have not been clarified over the years [17]. The role of autoimmunity against extravasated secretions from lobules has been advocated in its etiology [4,18]. The notion of IGM as an autoimmune disease is supported by positive responses observed in patients to steroid and immunosuppressive therapies, as well as extramammary manifestations such as erythema nodosum and arthritis [7,19]. Furthermore, an immunohistochemical study by Erhan et al. demonstrated T cell dominance in 14 out of 18 cases, suggesting an autoimmune pathophysiological process driven by reactive T cell-mediated inflammation and centrilobular granulomas against ductal damage [20]. The association between IGM and EN was first described by Adams et al., with the presence of EN alongside IGM serving as an indicator of autoimmunity in IGM’s etiology [21]. Sener et al. observed the presence of EN in 6 (17.1%) out of 37 IGM patients. They hypothesized that a hormonal etiology may be responsible, given that four of the six patients were pregnant. They proposed a link between increased estrogen production during pregnancy and heightened EN incidence [17]. However, in our study, none of seven cases of EN was pregnant, which suggests that hormonal conditions alone might not fully explain the etiology of EN. Cetin et al. reported higher rates of bilateral involvement and corticosteroid resistance in IGM patients with EN [8], which did not align with our findings. Only one of our seven patients with erythema nodosum exhibited bilateral involvement, one patient experienced recurrence, and two displayed steroid resistance. These rates were not significantly different from those observed in the entire IGM patient group.

Recent studies conducted in Turkey have highlighted the role of immune dysregulation in IGM. Ucaryilmaz et al. investigated the role of regulatory T and B cells’ involvement in the pathogenesis of IGM. Regulatory T cells (Tregs), a subset of CD4+ T cells, suppress the proliferation and cytokine production of active T cells. The study revealed notable alterations in Treg subgroups among active IGM and remission patients, implying Treg-specific anomalies in IGM patients [22].

Cytokines are pivotal proteins that stimulate the immune system, contributing to lymphocyte growth, differentiation, antigen elimination, and hematopoietic cell development. They also participate in inflammatory responses and wound healing. Several studies suggest that cytokines play an active role in autoimmunity [7]. For instance, interleukin-33 (IL-33), which has been the subject of study in rheumatological autoimmune diseases such as rheumatoid arthritis and systemic lupus erythematosus (SLE), has demonstrated increased levels and roles in cytokine synthesis and the inflammatory response [20,23]. Similarly, research conducted in our country examined the relationship between IL-33, IGM, and breast cancer, revealing significantly elevated IL-33 levels in both diseases compared to controls [24]. Proinflammatory cytokines IL-17, IL-22, and IL-23 were also examined by Saydam et al., who observed significant differences in IL-22 and IL-23 levels between IGM patients and controls. This suggests that autoimmunity may play a role in IGM’s etiopathogenesis [7]. Koksal et al. investigated the relationship between the proinflammatory cytokines including IL-4, IL-8, IL-17, TNF-α, and IGM. Their findings revealed that IGM patients exhibited significantly elevated levels of IL-8, IL-10, and IL-17 compared to controls. In that study, no significant difference was found in IL-4 and TNF-α levels [25]. Conversely, our study demonstrated significantly elevated IL-4 levels in active IGM patients when compared to controls (*p* = 0.003). Additionally, we postulate that these alterations in the IL-4 cytokine level, tied to allergic immune responses, underscore immune disorder and play a role in the etiopathogenesis of autoimmunity, as seen in other studies.

Beyond genetics, environmental factors significantly influence autoimmunity development, with recent years witnessing a surge in autoimmune disease incidence attributed to environmental triggers, particularly food and its components [11]. Based on this point, studies have been conducted to investigate the potential relationship between food intolerance and various diseases. For instance, a study involving rheumatoid arthritis patients revealed symptom regression in 91% of patients subjected to a restricted diet targeting their sensitivities, indicating the potential impact of dietary factors [26]. In a study by Cai et al., specific IgG antibody values against certain foods were identified in patients with Crohn’s disease and ulcerative colitis, suggesting a potential link between food intolerance and the development of these diseases. Egg is the most frequently identified food allergen in these patients [27]. Coucke et al. established a link between autoimmune diseases and food intolerance, identifying reactive food epitopes such as cow’s milk, casein, egg white, and wheat as potential triggers in autoimmune patients [11]. Interestingly, in our study, allergy to egg white was 31.25% in IGM patients compared to 62.5% in the normal cohort.

Both allergy and autoimmunity arise from compromised immune systems and involve local inflammation that damages target tissues. Until recently, these disease processes were considered to be quite distinct; however, new discoveries point to a possible pathogenetic link [28].

Histamine, a mediator in allergies, has been observed to increase in some autoimmune diseases. For example, histamine release rates were found to be elevated in patients with Crohn’s diseases, with levels correlating with disease activity [29]. Lee et al. demonstrated that the mast cell-deficient mice exhibited resistance to the development of rheumatoid arthritis, which suggests a potential role for mast cells in mediating the interaction between autoantibodies and inflammatory mediators in arthritis [30]. In the present study, histamine levels were found to be significantly higher in IGM patients (*p* < 0.001), suggesting a potential role for mast cells in the etiopathogenesis of IGM. Blocking histamine effects could hold therapeutic promise in treating IGM.

In the present study, we utilized IgG-based food intolerance testing across 54 foods in 32 active IGM patients and 32 healthy individuals. Over 40% of IGM patients demonstrated intolerance to lentils, wheat flour, rye flour, processed cheese, cow’s milk, egg whites, and curry, while similar rates of intolerance were observed in the healthy cohort. The prevalence of lentil and curry intolerances was significantly higher in the patient group than the control group. These findings indicate that lentils, which is a commonly consumed food in our society, may act as a triggering factor in the disease and should be considered in the conservative treatment approach.

Our study has several limitations. It is a single-center study representing only a single geographic region, and it assessed 54 food items that are more frequently consumed. Although the study design was suitable for G power analysis, the evaluation was conducted with small sample sizes across groups. Furthermore, the impact of dietary changes in response to food intolerances on the progression of the disease was not assessed, as it is planned to be the subject of a future study. However, the study was designed as a pilot, aiming to provide preliminary insights for future research with larger and more diverse cohorts. We believe that our findings can contribute to the growing body of knowledge on this topic and stimulate further studies with broader applicability.

## 5. Conclusions

The present study revealed a general similarity in food intolerance values across groups, with the exception of lentils and curry, which exhibited higher intolerance values in the IGM group. This observation leads us to speculate that lentils, a commonly consumed dietary item within our society, may potentially exert a triggering role in the manifestation of the disease and thereby hold significance within the context of a conservative treatment approach.

The renowned spice blend of Indian cuisine, curry, garnered attention due to its elevated intolerance values within the IGM group. Given the substantial prevalence of IGM in India, our findings could serve as a catalyst for further research into the potential impact of other dietary components, such as curry, on the etiological underpinnings of IGM—particularly within densely populated countries like India and China.

The elevated levels of histamine and IL-4 observed in IGM patients suggest a correlation with allergic and immunological factors. In light of these findings, we propose that further research is needed to explore the potential role of anti-histaminergic agents in the treatment of IGM patients with elevated histamine levels. This may offer an alternative therapeutic strategy, although the exact mechanistic involvement of these factors remains to be clarified.

## Figures and Tables

**Table 1:** Symptoms and clinical findings of IGM patients.

Symptoms and Clinical Findings	N (%)
Localization	
Right	9 (28.1)
Left	17 (53.1)
Bilateral	5 (15.6)
Mass	27 (84.4)
Pain	25 (78.1)
Flux	10 (31.2)
Erythema	9 (28.1)
Abscess	19 (59.4)
Fistula	14 (43.8)
Ulcers	2 (6.3)
Axillary lymphadenopathy	13 (40.6)
Recurrence	7 (21.8)
Steroid resistance	6 (18.75)

**Table 2:** Histamine, IL-4, IL-4R, and HRF in IGM and control groups.

	Patients			Control Group			
	Mean	s.s	Median	Mean	s.s	Median	*p*
Histamine (pg/mL)	6176.56	±1970.39	6180	3572.81	±1809.41	2940	**<0.001**
IL-4 (pg/mL)	15.36	±8.11	12.3	10.59	±3.96	10.2	**0.003**
Il-4R (pg/mL)	59.37	±7.1	61.04	59.64	±6.93	61.41	0.702
HRF (ng/mL)	10.69	±1.28	10.99	10.74	±1.25	11.06	0.702

Mann-Whitney U Test.

**Table 3:** Intolerance rates in IGM and control groups.

	IGM		Control				IGM		Control		
	n	%	n	%	*p*		n	%	n	%	*p*
Lentil	23	−71.9	14	−43.8	**0.023**	Pork	3	−9.38	2	−6.25	0.641
Egg white	10	−31.3	20	−62.5	0.012	White beans	3	−9.38	2	−6.25	0.641
Cow milk	13	−40.6	16	−50	0.451	Almond	2	−6.25	3	−9.38	0.641
Processed cheese	12	−37.5	12	−37.5	1	Hazelnut	1	−3.13	3	−9.38	0.302
Wheat flour	12	−37.5	9	−28.1	0.424	Banana	3	−9.38	0	0	0.076
Casein	9	−28.1	9	−28.1	1	Sourdough	1	−3.13	2	−6.25	0.554
Rye flour	11	−34.4	7	−21.9	0.266	Tuna	2	−6.25	0	0	0.151
Egg yolk	5	−15.6	11	−34.4	0.083	Chicken	1	−3.13	1	−3.13	1
Vanilla	10	−31.3	5	−15.6	0.14	Soybean	0	0	2	−6.25	0.151
Curry	11	−34.4	4	−12.5	**0.039**	Salmon	1	−3.13	1	−3.13	1
Garlic	4	−12.5	8	−25	0.2	Potato	0	0	2	−6.25	0.151
Fig	2	−6.25	7	−21.9	0.072	Carrot	0	0	2	−6.25	0.151
Oat flour	2	−6.25	4	−12.5	0.391	Green beans	0	0	1	−3.13	0.313
Cod fish	4	−12.5	2	−6.25	0.391	Orange	0	0	1	−3.13	0.313
Goat milk	1	−3.13	5	−15.6	0.086	Rice	1	−3.13	0	0	0.313
Gluten	3	−9.38	3	−9.38	1	Kiwi	0	0	1	−3.13	0.313
Barley flour	3	−9.38	3	−9.38	1	Mustard	1	−3.13	0	0	0.313
Peanut	2	−6.25	3	−9.38	0.641	Apple	1	−3.13	0	0	0.313

Chi-Square Test.

## Data Availability

The data underlying this article are available in the article. If needed, please contact the corresponding author. The email address is mugeyrdcn@hotmail.com.
